# Investigating Direct and Moderating Effects of Social Connectedness and Perceived Social Support on Suicidal Ideation in Older Adults With Depression: A Prospective Study

**DOI:** 10.1016/j.bpsgos.2025.100513

**Published:** 2025-04-21

**Authors:** Madison Stoms, Anna Szücs, Yanni Wang, Katalin Szanto, Hanga Galfalvy

**Affiliations:** a Department of Biostatistics, Columbia University, New York, New York; b Faculty of Behavioural and Movement Sciences, Vrije Universiteit Amsterdam, Amsterdam, the Netherlands; c Division of Family Medicine, Department of Medicine, Yong Loo Lin School of Medicine, National University of Singapore, Singapore; d Department of Epidemiology, University of California, Los Angeles, Los Angeles, California; e Department of Psychiatry, University of Pittsburgh, Pittsburgh, Pennsylvania; f Department of Psychiatry, Columbia University, New York, New York

**Keywords:** Old age, Physical illness, Protective factors, Social connectedness, Social support, Suicidal ideation

## Abstract

**Background:**

Maintaining one’s social capital may protect older adults with depression from contemplating suicide, possibly by contributing to overall well-being and mitigating the negative effects of arising difficulties such as worsening mental or physical health. However, it remains unclear whether such protective overall and mitigating effects stem primarily from the size and diversity of one’s social network (social connectedness) or from the feeling of being supported by others (perceived social support) and whether these effects persist over time.

**Methods:**

In a longitudinal sample of adults with depression ages ≥50 years (*N* = 287, mean age = 64 years, mean follow-up time = 2 years), with most participants having suicidal ideation (*n* = 203), zero-inflated negative binomial regression models were used to prospectively evaluate whether social connectedness and perceived social support measured at baseline decreased the presence and severity of suicidal ideation, and whether they moderated the unfavorable effect of baseline depression severity and physical illness on ideation presence and severity at baseline and during short- and long-term follow-ups.

**Results:**

In prospective models, both ideation presence and ideation severity decreased with social connectedness (ideation presence: odds ratio = 0.77, SE = 0.10, *p* = .003; ideation severity: rate ratio [RR] = 0.84, SE = 0.05, *p* = .005). Perceived social support only decreased ideation severity (RR = 0.64, SE = 0.05, *p* < .001). No moderation effect with social health measures reached significance.

**Conclusions:**

Social connectedness and perceived social support confer lasting protection against suicidal ideation. Clinicians should encourage preventive maintenance of diverse social networks in their middle-age and older patients/clients with depression and help them find adequate social support during acute suicidal crises.

## Plain Language Summary

This longitudinal study was conducted with adults ages ≥50 years with depression. These adults were followed up for up to 4 years (2 years on average). The study found that, over the follow-up period, social connectedness (i.e., the extent of one’s social network) decreased both the likelihood of suicidal thoughts and the intensity of such thoughts in individuals who were experiencing them. Perceived social support (the quality of one’s social interactions based on one's own estimation) also decreased the intensity of suicidal thoughts in those who were experiencing such thoughts. These findings were not influenced by participants’ level of depressive symptoms or physical illness.

Suicide risk tends to increase during older age, with approximately one-quarter of deaths by suicide (27.2%) occurring in adults age ≥60 years (1). Health-related risk factors, such as acute depression and physical illness, have been consistently associated with suicide risk in older age (2, 3, 4).

While not all suicide deaths are preceded by suicidal thoughts, we have previously shown that the risk of future suicidal behavior is highest in groups with persistently high and/or highly variable suicidal ideation (5). Moreover, suicidal ideation can be a debilitating mental health condition in and of itself (6). Thus, the study of ideation and its predictors in older adults has the potential to contribute not only to the reduction of suicide risk but also to the improvement of quality of life.

Mental and physical health do not explain suicidal ideation entirely given that not all older adults who have depression or are physically ill develop suicidal thoughts (7,8). The third pillar of health, social health, constitutes an established protective factor against suicide risk (9, 10, 11), which must be considered together with other aspects of health.

Social health has been defined as “the quantity and quality of an individual’s interpersonal ties and extent of involvement in the community” (12). Cohen and Wills proposed to organize its different components into measures of structure and function, with structure evaluating the extent of one’s social network, i.e., its quantity (henceforth, social connectedness) and function appraising the resources provided by the network, i.e., its quality (henceforth, perceived social support) (13). They found that social connectedness mostly had a positive main effect on well-being, whereas perceived social support mostly had a situational buffering effect on the negative impact of specific stressors (13). However, these results have not been replicated in the literature on aging, where social connectedness and perceived social support have been investigated separately or without testing their moderating effects on adverse events and other stressors (14). Studies that have tested associations of health and well-being in older adults with both social health constructs have yielded mixed results, finding stronger associations with either social connectedness (15) or perceived social support (16).

In our previous work, we found evidence that social connectedness and perceived social support were lower in suicide attempters and individuals with suicidal ideation than in both participants with depression and nonpsychiatric comparison participants ages ≥50 years (17). In the same study, executive dysfunction was linked to low social connectedness in attempters but high social connectedness in healthy comparison participants, suggesting that social connectedness may buffer the risk of suicidal behavior associated with certain forms of cognitive decline. However, such a moderating effect remains to be confirmed longitudinally and to be tested in the context of other, more prevalent risk factors that impact mental and physical health, namely depression and physical illness.

**Figure 1:**
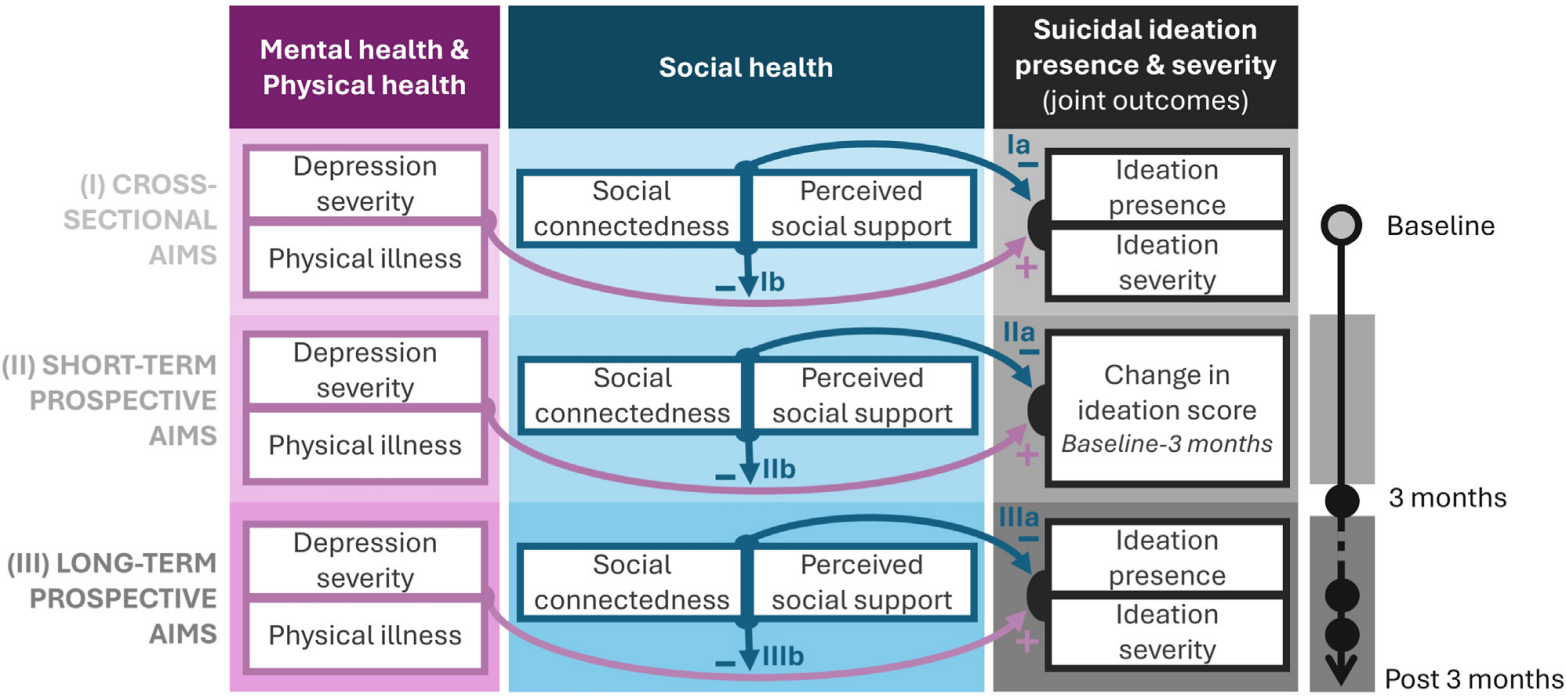
Graphical representation of study aims. Study aims are divided into 3 parts: cross-sectional aims and short- and long-term prospective aims. Arrows represent (Ia–IIIa) main effects (direct associations in the case of the cross-sectional aims) and (Ib–IIIb) moderating effects (differential associations in the case of cross-sectional aims) with “+” and “−” signs indicating the expected direction of each association.

Using the presence and severity of suicidal ideation as indicators of suicide risk in adults ages ≥50 years, in the current study, we aimed to investigate whether social connectedness and perceived social support 1) have protective effects (negative associations) against the presence and severity of suicidal ideation and/or 2) buffering (i.e., mitigating) effects on the positive association of depression severity and physical illness with suicidal ideation (Figure 1). The study tested these effects (aim Ia and Ib) at baseline (cross-sectional aims) and prospectively, (aim IIa and IIb) during the first 3 months and (aim IIIa and IIIb) later during follow-up (short-term and long-term prospective aims). Following Cohen and Wills’s theory (13), we expected to find evidence of stronger main effects with social connectedness and stronger buffering effects with perceived social support.

## Methods and Materials

### Participants

The study comprised 287 adults with depression ages ≥50 years (mean 63.66 years) enrolled in the Longitudinal Research Program in Late-Life Suicide at the University of Pittsburgh (18). To be eligible for the program, participants had to be ≥50 years of age, score >21 points on the Mini-Mental State Examination, have no diagnosis of dementia or bipolar disorder, and have no symptoms of intoxication or withdrawal from alcohol or drugs. Participants included in the current study had moderate to severe depression, defined as a formal diagnosis of a current depressive episode based on the Structured Clinical Interview for DSM-IV Axis I disorders (19) and a score of ≥14 points on the Hamilton Rating Scale for Depression (HRSD) (20) (see Measures). Of the total sample used in the current study, 203 participants reported some level of suicidal ideation upon recruitment, whereas 84 reported none (Table 1). Data used in this study were collected over a period of 18.5 years from October 2003 to April 2022.

**Table 1:** Participant Characteristics by Presence of Ideation at Baseline

	Full Sample, *N* = 287	Nonideators, *n* = 84	Ideators, *n* = 203	*p* Value
Age, Years	63.66 (7.77)	64.67 (8.15)	63.24 (7.59)	.300
Sex				>.900
Female	155 (54.01%)	45 (53.57%)	110 (54.19%)	
Male	132 (45.99%)	39 (46.43%)	93 (45.81%)	
Race				.300
Asian	1 (0.35%)	0 (0%)	1 (0.49%)	
Black or African American	48 (16.73%)	19 (22.62%)	29 (14.29%)	
Multiracial	1 (0.35%)	0 (0%)	1 (0.49%)	
White	237 (82.58%)	65 (77.38%)	172 (84.73%)	
Physical Illness, CIRS-G^a^	9.13 (4.39)	9.07 (4.35)	9.15 (4.41)	>.900
Depression Severity, HRSD^b^	19.29 (5.24)	17.61 (4.04)	19.99 (5.52)	<.001
Social Connectedness, SNI^c^				
Network diversity	4.16 (1.93)	4.55 (2.07)	4.00 (1.85)	.030
Embedded networks	0.95 (1.07)	1.20 (1.20)	0.84 (0.99)	.014
Number of people with interactions	9.63 (6.43)	11.10 (6.89)	9.03 (6.15)	.013
Composite score^d^	−0.06 (2.89)	0.62 (3.06)	−0.34 (2.77)	.012
Perceived Social Support, ISEL^e^				
Self-esteem	5.27 (2.89)	6.42 (2.88)	4.79 (2.76)	<.001
Belonging	6.58 (3.31)	7.52 (3.13)	6.18 (3.31)	.001
Appraisal	7.72 (2.90)	8.32 (2.81)	7.47 (2.91)	.020
Tangible support	7.39 (3.31)	8.12 (3.08)	7.08 (3.36)	.016
Composite score^d^	0.18 (2.44)	1.02 (2.26)	−0.18 (2.43)	<.001
Worst Lifetime Ideation, SSI^f^	15.92 (11.81)	2.45 (5.52)	21.45 (8.88)	<.001
Current Ideation, SSI^f^	12.53 (11.00)	0.00 (0.00)	17.69 (8.91)	<.001
Follow-Up Time, Months	25.43 (14.47)	22.13 (14.73)	26.80 (14.17)	.013

Values are presented as *n* (%) or mean (SD). *p* Values were obtained using Wilcoxon rank-sum tests for continuous variables and Pearson’s χ^2^ tests for categorical variables. Baseline values are presented for all measures except follow-up time.

CIRS-G, Cumulative Illness Burden-Geriatric version; HRSD, Hamilton Rating Scale for Depression; ISEL, Interpersonal Support Evaluation List; SNI, Social Network Index; SSI, Beck Scale of Suicidal Ideation.

a Range: 0 (physically healthy) to 52 points (life-threatening physical illness in all organ systems).

b Range: 0 to 47 points; score ≥ 14 points indicates moderate to severe depression.

c Range: 0 to 12 points for network diversity and embedded networks and 0 to 68 for network size.

d Composite scores were obtained by averaging the corresponding subscale scores.

e Subscale range: 0 to 12.

f Range: 0 to 38 points.

### Procedure

The authors assert that all procedures that contributed to this work comply with the ethical standards of the relevant national and institutional committees on human experimentation and with the Helsinki Declaration of 1975, as revised in 2013. All procedures involving human participants were approved by the University of Pittsburgh’s Institutional Review Board (Approval No. STUDY19060351).

Participants were recruited from psychiatric inpatient units, outpatient groups, depression clinics, private practices, and the university’s registry of research volunteers. They were asked to give written informed consent to participate in the study. After their baseline assessment, participants were assessed at a 3-month follow-up and then yearly for up to 4 years (mean follow-up time = 2 years) (see Sample Characteristics). Assessments were carried out by trained research assistants in person, over the phone, or by Zoom, according to participants’ preference. The interrater reliability of the HRSD was assessed regularly, and questions about assessments were discussed with the principal investigator and other study team members during weekly meetings.

### Measures

#### Baseline Assessments (Social Health Measures)

Social health measures were collected at baseline, together with basic demographic variables such as age and sex.

Social connectedness was measured with the Social Network Index (SNI) (21), which assesses 3 dimensions of connectedness in a clinician-administered questionnaire: 1) network diversity, the number of distinct networks wherein the participant has regular contacts with 1 person or more (e.g., as parent, child, friend); 2) network size, the total number of people with whom the participant has contact at least once every 2 weeks; and 3) number of embedded networks, the number of networks wherein the participant plays an active role (e.g., family, church, work, volunteering). Because the maximum number of networks in the questionnaire is set at 12, network diversity and number of embedded networks range from 0 to 12. For network size, the maximum score is 68, because the maximum number of people that can be reported is 1 for question 1 (spouse), 2 for questions 3 or 4 (parents and in-laws), and 7 for all other questions.

Perceived social support was measured with the Interpersonal Support Evaluation List (ISEL) (22), a self-report that assesses perceived social support with 4 subscales: 1) appraisal support, having people in one’s network whom one can ask for an objective opinion about intimate or sensitive matters; 2) tangible support, having people in one’s network who can provide practical help; 3) self-esteem support, having people in one’s network who are encouraging and bolster one’s self-esteem; and 4) belonging support, having people in one’s network with whom one can socialize and do activities. Each subscale is scored on 4-point Likert scales ranging from 0 to 4, with scores ranging from 0 to 12 for each subscale.

The subscales of SNI and ISEL were used to construct composite social health measures of social connectedness and perceived social support, respectively (see Statistical Analyses).

#### Other Assessments for Which Baseline Scores Were Used (Mental and Physical Health Measures)

We used baseline values of depression severity and physical illness in all analyses.

Depression severity was measured with the 17-item HRSD (20), a clinician-administered semistructured questionnaire. Items were evaluated on a scale between 0 and 5 or 0 and 3 points. The item that assesses suicidal ideation (item 3) was not counted in the total scores, which therefore ranged between 0 and 47, with scores of ≥14 indicating moderate to severe depression.

Physical illness was assessed using the Cumulative Illness Rating Scale-Geriatric version (23), a clinician-administered tool where each diagnosed physical illness of the participant is rated on illness severity for 14 organ systems. For each organ system, illness severity ranges from 0 (no health issue) to 4 (extremely severe/life-threatening/terminal condition). The psychiatric illness category was excluded from total scores. The rest of the organ system scores were added to create a total score indicating physical illness severity ranging from 0 (physically healthy) to 52 points (highest possible physical illness severity in all organ systems).

#### Longitudinal Assessment of the Outcome Measure (Suicidal Ideation)

Suicidal ideation was measured using the Beck Scale of Suicidal Ideation (24) at baseline, 3 months, and then yearly for the rest of the follow-up. The scale is clinician administered and assesses the severity of suicidal contemplation and planning. It comprises 19 items rated on a 3-point scale between 0 and 2, with 2 representing highest severity. Total scores range from 0 to 38 points, with 0 corresponding to no suicidal ideation and other values to some degree of suicidal ideation. Scores were computed for both current and worst ideation since last assessment. We used current ideation between follow-up assessments as our primary longitudinal data points (5). Worst ideation data points are included in the Supplement as an alternative analysis.

### Statistical Analyses

The 2 social health measures of interest, social connectedness and perceived social support, were obtained by averaging the subscales of SNI and ISEL, respectively. Before combining the subscales, the measures were scaled and outliers (representing 6.3% of values) were winsorized. The 2 health-related risk factors of interest, physical illness and depression severity, were centered and scaled prior to model fitting.

The statistical analysis was performed in 3 stages to match the study’s cross-sectional and short- and long-term prospective aims (Figure 1). Separate models were fit with social connectedness and perceived social support. All models covaried for age and sex. Given that 6 independent regression models were conducted, testing the effects of the 2 social health measures at 3 different time intervals (Figure 1), we considered a Bonferroni-corrected threshold for significance at *p* < .01 (rounded up from *p* < .008 for ease of interpretation).

#### Cross-Sectional Analysis (Aim I)

At baseline, the proportion of nonideators in the sample was close to 30%, and the distribution of ideation scores showed evidence of overdispersion, i.e., greater variability than expected (Table 1 and Figure S1); thus, zero-inflated negative binomial regression models were used (Table 2). This form of regression, developed for count data with zeros exceeding the expected proportion, consists of 2 linked models that assess the odds of zero ideation (ideation absence; reported in terms of ideation presence for ease of clinical interpretation) and the severity of nonzero ideation (ideation severity among participants who have ideation), respectively. Negative binomial regression has previously been used in the literature to model suicidal ideation data (25), including ideation scores measured by the Beck Suicidal Ideation Scale, similar to here (26). We report estimates as odds ratios (ORs) for the presence of ideation (zero-inflated component) and as rate ratios (RRs) for severity of ideation among ideators (severity of nonzero ideation component), each time with 95% CIs. Both ORs and RRs were calculated by exponentiating the respective model coefficients. The estimates of primary interest were those of social connectedness and perceived social support (aim Ia) and their interactions with depression severity and physical illness (aim Ib).

#### Prospective Analysis (Aims II and III)

Prospective ideation scores showed a steep decrease in the first 3 months after baseline, followed by a slight re-increase and then stabilization in most cases (Figure S2). Because many participants were recruited as inpatients, this pattern may correspond to the intensification of psychiatric treatment during a suicidal crisis and is consistent with observations in younger age groups (27). Two sets of models were fit to test (aim II) short-term and (aim III) long-term trends in suicidal ideation. To evaluate both (aims IIa and IIIa) main effects and (aims IIb and IIIb) interaction effects, variables were entered in a progressive manner, with the social health measure of interest (social connectedness or perceived social support) and demographic covariates (age and sex) being entered first, the 2 health-related risk factors (depression severity and physical illness) being entered second, and interactions of depression severity and physical illness with the social health measure of interest being entered last.

#### Prospective Analysis: Short-Term Prediction (Aim II)

Robust linear regression models predicted change in suicidal ideation from baseline to 3 months (Table 3). Change scores were obtained by subtracting the baseline current ideation score from the 3-month score, such that positive values indicate worsening ideation from baseline to 3 months. Robust models were necessary due to extreme skewness in the change scores for ideation. Participants whose 3-month visit was delayed to 6 months (180 days) or more after study entry were excluded from this analysis, thereby decreasing the analysis set from 287 to 245 participants.

#### Prospective Analysis: Long-Term Prediction (Aim III)

Mixed-effects zero-inflated negative binomial models were used to predict longitudinally measured ideation scores, starting at the 3-month follow-up visit, or later if that was not possible, and including all subsequent follow-up assessments of suicidal ideation. Participant-specific random intercept modeled the within-participant correlations of repeated measures. Time (in days) elapsed since the baseline assessment was log-transformed and included as a covariate. Repeated measures of ideation have previously been analyzed using a negative binomial regression model via generalized estimating equation analysis (25). Similar to the cross-sectional models, these models jointly tested the predictors for the likelihood of zero suicidal ideation (ideation presence) and the severity of nonzero ideation (ideation severity). Current ideation at each assessment was used as the outcome in the primary analysis (Table 4), while worst ideation since the last assessment was used in alternative models (Table S2).

#### Sensitivity Analysis

Given the documented differences in the association of social and mental health between middle-age and older males and females (28), the robustness of our main findings was tested in secondary models covarying for the interaction between social health measures and sex (Tables S4–S6). These models only retained other interaction terms if they reached at least a nominal level of significance (i.e., *p* < .05) in the principal analysis.

## Results

### Sample Characteristics

Participant characteristics are summarized in Table 1. The mean age was 63.66 years (SD = 7.77), 54.01% were female (*n* = 155), and 82.58% were White (*n* = 237). The average follow-up time was 25.43 months (SD = 14.47, range = 0–43.20). On average, ideators were followed for approximately 5 months longer than nonideators (see Discussion). At baseline, 203 participants (70.73%) reported some level of suicidal ideation. Physical illness severity did not differ between ideators and nonideators. Ideators were more severely depressed than nonideators and reported higher scores on average for their ideation level at baseline. Pairwise correlations between study variables are shown in Table S1.

**Figure 2:**
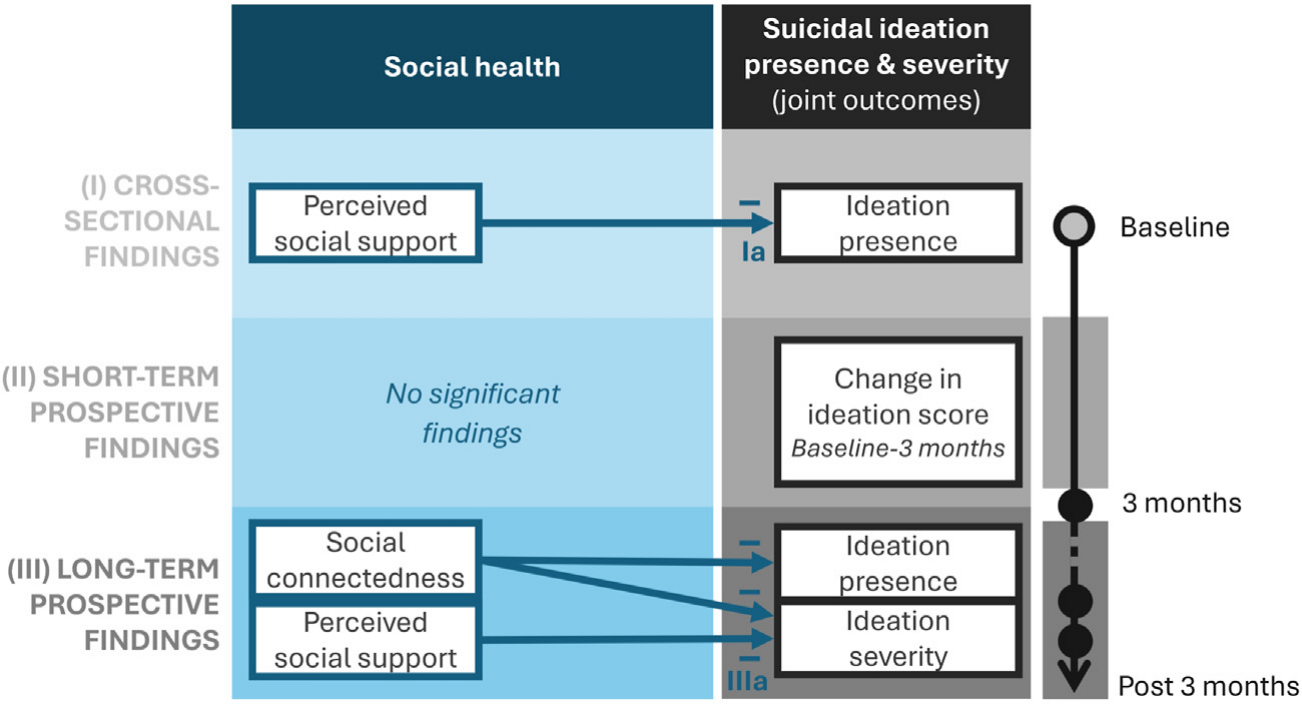
Graphical representation of main findings. Main findings are numbered I and III to match the corresponding study aims (see Figure 1 for all study aims). Arrows represent main effects (direct associations in the case of the cross-sectional aims) with “−” signs indicating negative (protective) associations.

A graphical summary of main findings is presented in Figure 2.

### Cross-Sectional Associations of Social Connectedness and Perceived Social Support With Suicidal Ideation Presence and Severity (Aim I)

Regression results for aim I are presented in Table 2.

**Table 2:** Cross-Sectional Effects of Social Connectedness and Perceived Social Support on Current Suicidal Ideation at Baseline

	Model Series 1: Social Health Measure = Social Connectedness						Model Series 2: Social Health Measure = Perceived Social Support					
	Model 1-1		Model 1-2		Model 1-3		Model 2-1		Model 2-2		Model 2–3	
	Ideation Presence	Ideation Severity	Ideation Presence	Ideation Severity	Ideation Presence	Ideation Severity	Ideation Presence	Ideation Severity	Ideation Presence	Ideation Severity	Ideation Presence	Ideation Severity
Social Health Measure^a^	0.896∗ (0.051)	0.973 (0.017)	0.899∗ (0.052)	0.97 (0.017)	0.877∗∗ (0.056)	0.971 (0.017)	0.811∗∗ (0.074)	0.977 (0.02)	0.833∗∗ (0.074)	0.977 (0.02)	0.812∗∗ (0.08)	0.975 (0.02)
Age	0.98 (0.017)	0.999 (0.006)	0.99 (0.018)	1.003 (0.006)	0.988 (0.018)	1.003 (0.006)	0.99 (0.017)	1 (0.006)	0.998 (0.018)	1.004 (0.006)	0.996 (0.018)	1.003 (0.006)
Sex, Male vs. Female	0.946 (0.283)	1.122 (0.104)	0.99 (0.276)	1.147 (0.105)	0.931 (0.297)	1.148 (0.106)	0.903 (0.301)	1.11 (0.104)	0.938 (0.259)	1.128 (0.105)	0.922 (0.302)	1.106 (0.103)
Depression Severity	–	–	1.091∗∗ (0.026)	1.017∗ (0.008)	1.101∗∗∗ (0.026)	1.018∗∗ (0.009)	–	–	1.083∗∗ (0.026)	1.016 (0.009)	1.094∗∗ (0.028)	1.017∗ (0.009)
Physical Illness	–	–	0.991 (0.032)	0.983 (0.01)	0.998 (0.033)	0.981 (0.011)	–	–	0.994 (0.032)	0.985 (0.01)	1.005 (0.033)	0.983 (0.01)
Social Health Measure^a^ × Depression Severity	–	–	–	–	0.984 (0.01)	1.001 (0.003)	–	–	–	–	0.983 (0.014)	1.004 (0.004)
Social Health Measure^a^ × Physical Illness	–	–	–	–	0.988 (0.011)	0.998 (0.004)	–	–	–	–	0.988 (0.015)	0.993 (0.005)

Estimates are presented as OR (SE) for ideation presence (zero inflation part) and RR (SE) for ideation severity and are coded such that ORs and RRs <1 indicate protective effects. For each social health measure, a series of 3 zero-inflated negative binomial models are presented (1-1 to 1-3 for social connectedness and 2-1 to 2-3 for perceived social support) predicting current suicidal ideation presence and severity at baseline. The threshold for significance is set to *p* < .01. Both series of 3 models correspond to progressive model results with health-related risk factors entered second and interactions between health-related risk factors and social health measures entered last.

∗*p* < .05 (nominal effect), ∗∗*p* < .01 (significant effect), ∗∗∗*p* < .001 (significant effect).

OR, odds ratio; RR, rate ratio.

a Social health measure corresponds to social connectedness in models 1-1 to 1-3 and to perceived social support in models 2-1 to 2-3.

#### Cross-Sectional Main Effects (Aim Ia)

Perceived social support was negatively associated with the presence of ideation at baseline (OR = 0.81; 95% CI, 0.72–0.91; *p* = .001) (Table 2, Model 2-1), and this association remained significant after controlling for depression severity and physical illness (Table 2, Model 2-2). Social connectedness had a weak association with the presence of ideation at baseline, which was not significant at the *p* < .01 cutoff.

#### Cross-Sectional Interaction Effects (Aim Ib)

Effects of depression severity and physical illness on the presence and severity of ideation did not vary with either social health measure (i.e., there were no significant interactions) (Table 2, Models 1-3 and 2-3).

### Prospective Effects of Social Connectedness and Perceived Social Support on Change in Suicidal Ideation Scores From Baseline to 3 Months (Aim II)

Regression results for Aim II are presented in Table 3. This analysis only comprised participants with a completed first follow-up assessment between 3 and 6 months (*n* = 245).

**Table 3:** Change in Suicidal Ideation Score During the First 3 Months Post Baseline

	Model Series 1: Social Health Measure = Social Connectedness			Model Series 2: Social Health Measure = Perceived Social Support		
	Model 1-1	Model 1-2	Model 1-3	Model 2-1	Model 2-2	Model 2-3
Social Health Measure^a^	−0.248 (0.252)	−0.265 (0.251)	−0.295 (0.251)	−0.321 (0.313)	−0.194 (0.309)	−0.195 (0.299)
Age	−0.013 (0.096)	0.045 (0.097)	0.049 (0.097)	−0.010 (0.098)	0.039 (0.097)	0.024 (0.094)
Sex, Male vs. Female	2.488 (1.501)	2.625 (1.474)	2.513 (1.474)	2.252 (1.510)	2.396 (1.465)	2.264 (1.416)
Depression Severity	–	0.344∗ (0.142)	0.334∗ (0.143)	–	0.365∗ (0.143)	0.364∗∗∗ (0.138)
Physical Illness	–	−0.276 (0.171)	−0.292 (0.171)	–	−0.250 (0.166)	−0.227 (0.161)
Social Health Measure^a^ × Depression Severity	–	–	−0.039 (0.049)	–	–	−0.027 (0.061)
Social Health Measure^a^ × Physical Illness	–	–	−0.061 (0.056)	–	–	−0.176∗ (0.071)

Values are presented as *b* coefficient (SE). For each social health measure, a series of 3 robust linear regression models are presented (1-1 to 1-3 for social connectedness and 2-1 to 2-3 for perceived social support) predicting change in current suicidal ideation score between baseline and 3-month follow-up. The threshold for significance is set to *p* < .01. Both series of 3 models correspond to progressive model results with health-related risk factors entered second and interactions between health-related risk factors and social health measures entered last. Positive regression coefficients indicate a risk-increasing effect on suicidal ideation.

∗*p* < .05 (nominal effect), ∗∗∗*p* < .001 (significant effect).

a Social health measure corresponds to social connectedness in models 1-1 to 1-3 and to perceived social support in models 2-1 to 2-3.

**Table 4:** Prediction of Current Suicidal Ideation by Social Connectedness and Perceived Social Support Post 3 Months

	Model Series 1: Social Health Measure = Social Connectedness						Model Series 2: Social Health Measure = Perceived Social Support					
	Model 1-1		Model 1-2		Model 1-3		Model 2-1		Model 2-2		Model 2–3	
	Ideation Presence	Ideation Severity	Ideation Presence	Ideation Severity	Ideation Presence	Ideation Severity	Ideation Presence	Ideation Severity	Ideation Presence	Ideation Severity	Ideation Presence	Ideation Severity
Time in Days, Log-Transformed	0.826 (0.285)	0.915 (0.889)	0.733 (0.408)	0.935 (0.069)	0.47 (0.889)	0.931 (0.068)	0.807 (0.31)	0.918 (0.069)	0.38*∗* (1.089)	0.938 (0.068)	0.554 (0.731)	0.912 (0.068)
Social Health Measure^a^	0.763∗∗∗ (0.097)	0.84∗∗ (0.053)	0.767∗∗ (0.114)	0.836∗∗ (0.054)	0.446 (1.914)	0.813∗∗∗ (0.049)	0.923 (0.127)	0.641∗∗∗ (0.046)	1.242 (0.137)	0.642∗∗∗ (0.044)	1.367 (0.311)	0.664∗∗∗ (0.047)
Age	0.971 (0.026)	0.959 (0.022)	0.97 (0.03)	0.967 (0.022)	0.903 (0.064)	0.961 (0.021)	0.986 (0.027)	0.98 (0.021)	0.837∗∗ (0.074)	0.986 (0.02)	0.921 (0.064)	0.989 (0.021)
Sex, Male vs. Female	0.962 (0.453)	0.592 (0.199)	0.616 (0.906)	0.685 (0.231)	0.272 (3.577)	0.676 (0.222)	0.564 (0.737)	0.664 (0.211)	0.137∗∗ (5.635)	0.725 (0.221)	0.173 (7.488)	0.679 (0.212)
Depression Severity	–	–	0.952 (0.056)	1.089∗∗ (0.035)	0.891 (0.168)	1.093∗∗∗ (0.035)	–	–	0.784∗∗∗ (0.094)	1.058 (0.031)	0.740*∗* (0.199)	1.068*∗* (0.033)
Physical Illness	–	–	1.189 (0.12)	0.98 (0.038)	5.055∗∗∗ (0.108)	0.948 (0.038)	–	–	4.532∗∗∗ (0.091)	0.963 (0.034)	1.982 (0.177)	0.976 (0.036)
Social Health Measure^a^ × Depression Severity	–	–	–	–	0.966 (0.046)	0.997 (0.011)	–	–	–	–	1.012 (0.039)	0.997 (0.015)
Social Health Measure^a^ × Physical Illness	–	–	–	–	0.9 (0.15)	0.979 (0.013)	–	–	–	–	1.224*∗* (0.08)	0.992 (0.018)

Estimates are presented as OR (SE) for ideation presence (zero inflation part) and RR (SE) for ideation severity and are coded such that ORs and RRs <1 indicate protective effects. For each social health measure, a series of 3 zero-inflated negative binomial models are presented (1-1 to 1-3 for social connectedness and 2-1 to 2-3 for perceived social support) predicting prospective current suicidal ideation presence and severity since the last assessment. The threshold for significance is set to *p* < .01. Both series of 3 models correspond to progressive model results with health-related risk factors entered second and interactions between health-related risk factors and social health measures entered last.

∗*p* < .05 (nominal effect), ∗∗*p* < .01 (significant effect), ∗∗∗*p* < .001 (significant effect).

OR, odds ratio; RR, rate ratio.

a Social health measure corresponds to social connectedness in models 1-1 to 1-3 and to perceived social support in models 2-1 to 2-3.

Neither social health measure had a direct effect on change in ideation at the *p* < .01 cutoff for significance or moderated the effect of depression severity or physical illness on suicidal ideation scores.

### Prospective Effects of Social Connectedness and Perceived Social Support on Suicidal Ideation Presence and Severity Beyond 3 Months (Aim III)

Regression results for aim III are presented in Table 4.

#### Long-Term Main Effects (Aim IIIa)

Social connectedness decreased both the presence and the severity of suicidal ideation prospectively (ideation presence: OR = 0.77; 95% CI = 0.65–0.91; *p* = .003; ideation severity: RR = 0.84; 95% CI, 0.74–0.95; *p* = .005) (Table 4, Model 1-1). Perceived social support only decreased ideation severity (RR = 0.64; 95% CI, 0.56–0.73; *p* < .001) (Table 4, Model 2-1). These effects were also present in alternative models using worst ideation as the outcome, where perceived social support additionally decreased ideation presence (Table S2).

#### Long-Term Buffering Effects (Aim IIIb)

There were no moderating effects of social connectedness or perceived social support either in main models predicting current suicidal ideation or in alternative models predicting worst suicidal ideation (Table S2).

### Sensitivity Analysis

All main findings for aims I and III were robust to controlling for potential interactions between social health and sex (Tables S3 and S4).

## Discussion

In a sample of adults ages ≥50 years with depression followed for an average of 2 years, we investigated the direct and buffering effects of 2 dimensions of social health on suicidal ideation. We found that social connectedness had protective prospective effects on both the presence and severity of suicidal ideation in the long term, whereas perceived social support was associated with less ideation presence at baseline and decreased ideation severity prospectively. These associations remained significant after controlling for depression severity and physical illness.

These findings suggest that social health confers lasting protection from suicide risk in the second half of life independently of depression severity or physical illness. Previous research has linked perceived social support to a lower likelihood of suicidal ideation (29,30). Although not contradicting these results, our study lends stronger prospective evidence to the notion that the extent of social capital may be protective. This is consistent with cross-sectional evidence that has shown associations of community participation (31,32) and overall social connectedness (33,34) with lower risk of suicidal ideation and behavior in older adults.

Previous longitudinal studies on social health and suicide risk have focused mainly on the likelihood of ideating (29,30) or dying by suicide (4) [for a review, see (9)]. Our results add to these findings by suggesting a protective effect of social connectedness and perceived social support on the severity of ideation in individuals who already experience suicidal thoughts.

Thus, improving any aspect of social health could work to reduce suicidal ideation and suicide risk in middle-age and older adults, as we have suggested elsewhere (35). Consistently, individuals who built a suicide crisis response plan that included social connections have been found more likely to seek social support from their network and to experience lower levels of ideation severity than control participants who received standard treatment (36). However, another pilot intervention that targeted social health did not demonstrate effects on suicidal ideation over 10 weeks, although it improved depression symptoms and self-reported quality of life (37). Randomized-controlled evidence in older adults remains insufficient to draw precise recommendations. Taken together with results in younger populations (10), it nonetheless speaks in favor of early, individually tailored interventions on social health during suicidal crises.

As supported by the moderate correlation (*r* = 0.46) between social connectedness and perceived social support in our sample (Table S1), these 2 social health measures may partially overlap. By definition, social connectedness and perceived social support both decrease social isolation, which is an important risk factor for suicide and is especially prominent during aging given the loss of relationships and bereavement typical of later life (38). Aging impacts social contacts in multiple ways in addition to the loss of loved ones, for example by increasing the likelihood of disability, financial difficulties, and loss of autonomy in oneself and one’s same-aged social circle (39). Aging tends to decrease informal social contacts (e.g., with friends or relatives) but may increase formal ones, such as community activities (40). However, according to initial cross-sectional evidence, informal contacts may have a stronger negative association with suicidal ideation than formal ones (41), suggesting that certain aging-related needs require specific forms of social relationships, beyond generally high social connectedness.

However, our study did not provide evidence for any buffering effect of social health measures on the risk of suicidal ideation associated with higher depression severity or physical illness. This suggests that the protective role of social health may not be dependent on the context of one’s mental and physical health. However, it is worth highlighting that our study only assessed the effects of baseline health measures on prospectively measured suicidal ideation. To fully evaluate the interplay between social, mental, and physical health, future research will need to test these moderating effects while accounting for changes in all factors over time.

This study’s strengths include its large subsample of individuals with suicidal ideation; prospective design; combined analysis of both presence and severity of ideation with the same statistical test, namely zero-inflated negative binomial regression; and a relatively stringent significance cutoff that reflects correction for multiple testing, which enabled a more robust interpretation of findings despite investigating 2 social health measures of interest at 3 time points. This is the first study to investigate the moderating effects of social connectedness and perceived social support on physical and mental health–related variables prospectively in mid- and late life.

With respect to the study’s limitations, we note that suicide risk was only assessed through suicidal ideation, without differentiating participants who had also engaged in suicidal behavior from individuals who had not. While some data on prospective suicidal behavior exists for these participants, statistical power was too low to test the protective and moderating effects of social health measures on suicidal behavior. The fact that nonideators were followed for a shorter time than ideators raises the issue that if they had been followed for the same amount of time, ideation might have been observed, potentially leading to the misclassification of some nonideators as ideators. We did not collect social health data during follow-up and thus could not test the effect of change in social health on suicidal ideation risk. Similarly, we did not have systematic baseline data on inpatient/outpatient status, which might have influenced social health and suicidal ideation. Furthermore, although our study covaried for age, it could not take into account environmental stressors typically encountered at different life stages, such as during working years versus after retirement (42). Our data collection spanned close to 2 decades, potentially resulting in shifts in social interaction needs and types between age cohorts, e.g., online versus in-person contacts (43).

### Conclusions

Our study’s findings encourage clinicians to consider their patients’ or clients’ social health when evaluating a suicidal crisis and to target social isolation early during treatment at any stage of a patient’s/client’s suicidal trajectory. Public prevention strategies that promote the conservation of social capital from early on and throughout the aging process could prevent the occurrence of suicidal ideation in the first place, while increasing well-being and quality of life more generally. Such strategies may include city planning that promotes aging-friendly built environments (44); social funds that ensure the availability of affordable and accessible leisure activities for older adults (45); and education programs to raise awareness about available sources of social support in vulnerable older populations (46). In the United States, formal social connectedness has increased in later age cohorts of older adults (40), suggesting an initial positive impact of ongoing measures and thereby advocating for stepping up such efforts. Future research needs to confirm the effects of social connectedness and perceived social support on suicidal ideation in other populations, cultures, and age cohorts and in the context of different life stressors to better inform clinical practice and policy change.

## Supplementary Material

Figures S1–S2 and Tables S1–S4

Key Resources Table
