# Supplementary material for: Investigating Direct and Moderating Effects of Social Connectedness and Perceived Social Support on Suicidal Ideation in Older Adults With Depression: A Prospective Study
Source: Biol Psychiatry Glob Open Sci. 2025 Apr 21;5(4):100513. doi: 10.1016/j.bpsgos.2025.100513 (PMC12166375; doi:10.1016/j.bpsgos.2025.100513)
Supplement: Figures S1–S2 and Tables S1–S4 [file mmc1.pdf]

SUPPLEMENTARY INFORMATION

Investigating Direct and Moderating Effects of Social Connectedness and Perceived Social Support on Suicidal Ideation in Depressed Aging Adults: A Prospective Study

Stoms *et al.*

Table S1 – *Correlation matrix of study variables (N = 287)*

	1	2	3	4	5
**1. Baseline Ideation**	1.00				
**2. Social Connectedness**	-0.18*	1.00			
**3. Perceived Social Support**	-0.22**	0.46**	1.00		
**4. Physical Illness**	-0.04	-0.18*	-0.07	1.00	
**5. Depression Severity**	0.25**	-0.07	-0.22**	0.03	1.00

Note. Coefficients indicate Pearson's r; * p<.05; ** p<.01

Figure S1 – *Distribution of current ideation levels at baseline*

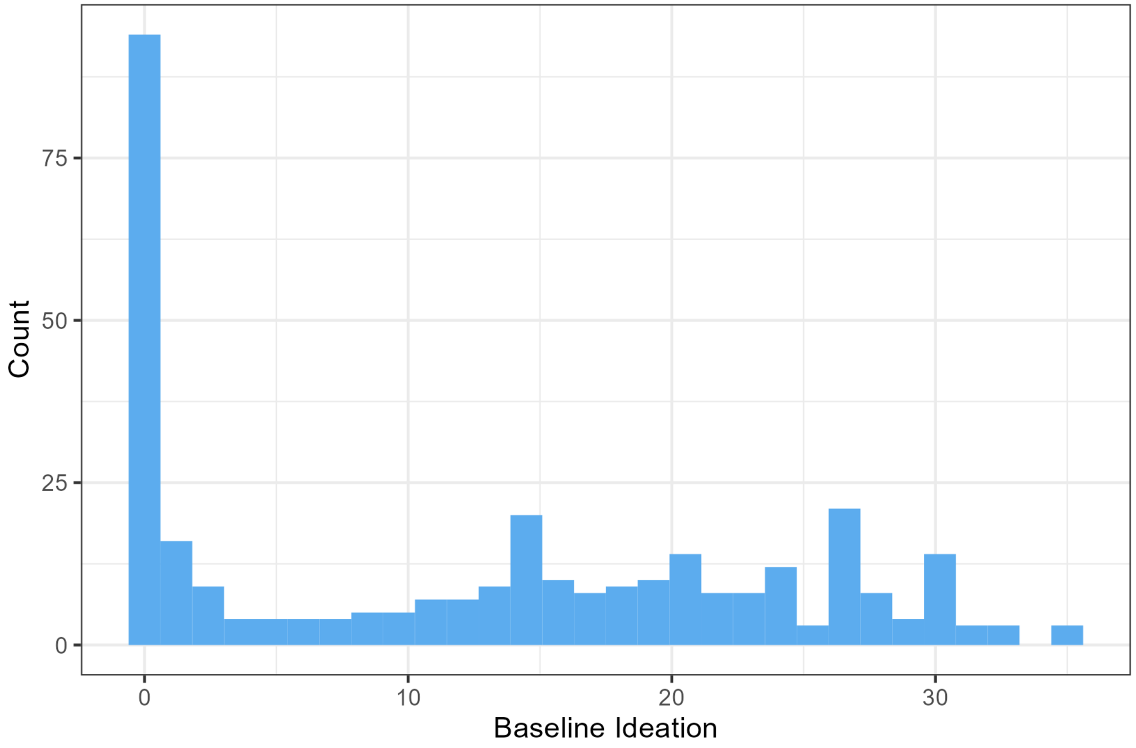

Figure S2 – *Longitudinal trends in current and worst suicidal ideation score*

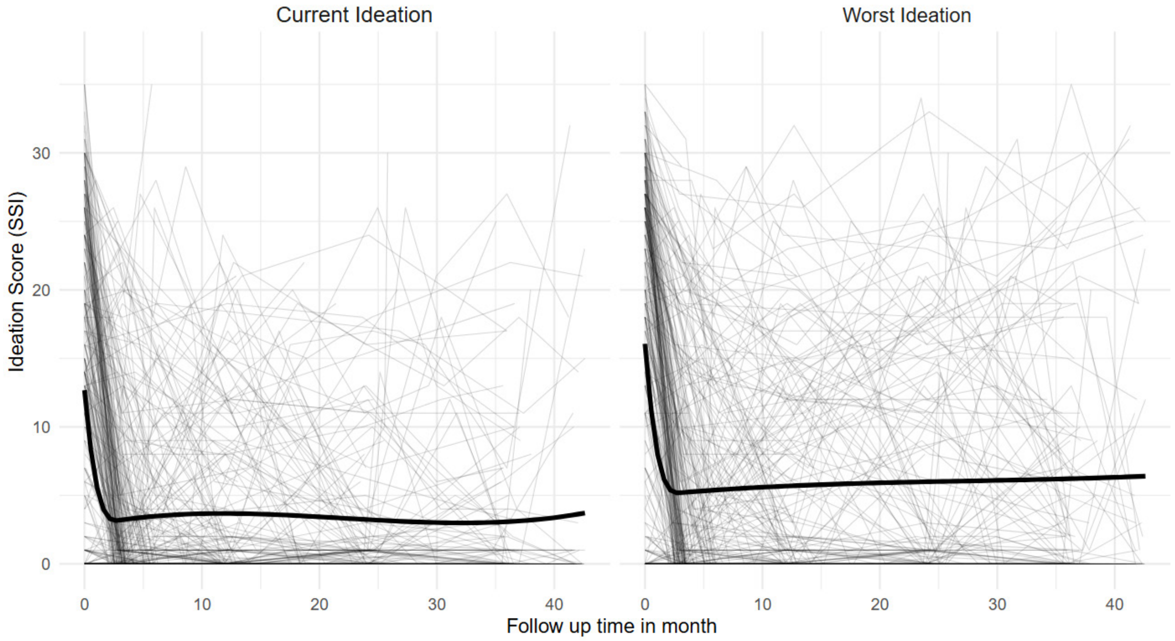

Note. Current ideation corresponds to suicidal ideation severity experienced at the time of the assessment and worst ideation to the worst suicidal ideation severity experienced since last assessment. Legend: SSI, Beck Scale of Suicidal Ideation.

Table S2

Prediction of worst suicidal ideation by social connectedness and perceived social support post-three months (alternative models to principal models with current ideation as outcome presented in Table 4)

	Model Series 1: **Social Health Measure = Social Connectedness**						Model Series 2: **Social Health Measure = Perceived Social Support**					
Dependent Variable	Prospectively Measured Worst Suicidal Ideation											
Model	Model 1-1		Model 1-2		Model 1-3		Model 2-1		Model 2-2		Model 2-3	
Model Component	Ideation Presence OR (SE)	Ideation Severity RR (SE)	Ideation Presence OR (SE)	Ideation Severity RR (SE)	Ideation Presence OR (SE)	Ideation Severity RR (SE)	Ideation Presence OR (SE)	Ideation Severity RR (SE)	Ideation Presence OR (SE)	Ideation Severity RR (SE)	Ideation Presence OR (SE)	Ideation Severity RR (SE)
Time in days (log-transformed)	0.733 (0.238)	0.979 (0.046)	0.708 (0.251)	0.981 (0.046)	0.707 (0.253)	0.981 (0.046)	0.751 (0.224)	0.975 (0.045)	0.736 (0.233)	0.976 (0.045)	0.735 (0.233)	0.976 (0.045)
#**Social Health Measure**	0.842** (0.059)	0.834* (0.049)	0.83** (0.066)	0.842* (0.050)	0.836* (0.067)	0.839* (0.050)	0.761* (0.104)	0.688* (0.05)	0.745* (0.112)	0.701* (0.051)	0.752* (0.127)	0.7* (0.051)
Age	0.973 (0.02)	0.934* (0.02)	0.962 (0.022)	0.946• (0.021)	0.965 (0.023)	0.945• (0.021)	0.983 (0.02)	0.958• (0.02)	0.969 (0.02)	0.966 (0.021)	0.969 (0.021)	0.967 (0.021)
Sex (Male vs Female)	0.739 (0.397)	0.692 (0.223)	0.628 (0.479)	0.759 (0.243)	0.613 (0.499)	0.731 (0.235)	0.666 (0.42)	0.721 (0.221)	0.577 (0.505)	0.771 (0.237)	0.581 (0.521)	0.775 (0.239)
Depression Severity	-	-	0.925* (0.031)	1.097* (0.034)	0.936• (0.034)	1.094* (0.034)	-	-	0.926* (0.03)	1.066• (0.032)	0.925* (0.031)	1.069• (0.032)
Physical Illness	-	-	1.069 (0.036)	0.98 (0.036)	1.071 (0.036)	0.977 (0.037)	-	-	1.076• (0.033)	0.984 (0.034)	1.077• (0.034)	0.983 (0.035)
#**Social Health Measure***Depression Severity	-	-	-	-	0.993 (0.009)	0.991 (0.011)	-	-	-	-	0.997 (0.014)	1.013 (0.014)
#**Social Health Measure***Physical Illness	-	-	-	-	1.001 (0.012)	0.992 (0.013)	-	-	-	-	1.004 (0.023)	1 (0.016)

Note. For each social health measure, a series of three zero-inflated negative binomial models are presented (numbered 1-1 to 1-3 for social connectedness and 2-1 to 2-3 for perceived social support) predicting prospective worst suicidal ideation presence and severity since last assessment. The threshold for significance is set to p <.01. Both series of three models correspond to progressive model results with health-related risk factors entered second and interactions between health-related risk factors and social health measures entered last. Estimates are presented as odds ratios for ideation presence (zero inflation part) and rate ratios for ideation severity and are coded such that odds ratios and rate ratios smaller than 1 indicate protective effects. Legend: OR, Odds Ratio; RR, Rate Ratio; SE, Standard Error; #, Social Health Measure corresponds to Social Connectedness in Models 1-1 to 1-3 and to Perceived Social Support in Models 2-1 to 2-3; •, p<.05 (nominal effect); significant effects: *, p<.01; **, p<.001.

Table S3

Cross-sectional zero-inflated models evaluating the effect of, respectively, social connectedness and perceived social support by sex *on current suicidal ideation at baseline (sensitivity analysis of principal models presented in Table 2).*

	Sensitivity Analysis Model 1: **Social Health Measure = Social Connectedness**		Sensitivity Analysis Model 2: **Social Health Measure = Perceived Social Support**	
Dependent Variable	Current Suicidal Ideation at Baseline			
Model Component	Ideation Presence Odds Ratio (SE)	Ideation Severity Rate Ratio (SE)	Ideation Presence Odds Ratio (SE)	Ideation Severity Rate Ratio (SE)
[#]**Social Health Measure**	.828* (0.077)	0.957 (0.023)	0.75** (0.117)	0.966 (0.026)
Age	0.989 (0.018)	1.003 (0.006)	0.999 (0.018)	1.004 (0.006)
Sex (Male vs Female)	0.947 (0.292)	1.158 (0.107)	0.812 (0.359)	1.133 (0.105)
Depression Severity	1.096* (0.026)	1.018[•] (0.008)	1.084* (0.026)	1.016 (0.009)
Physical Illness	0.997 (0.032)	0.983 (0.01)	0.998 (0.032)	0.985 (0.01)
[#]**Social Health Measure***Sex (Male vs Female)	1.197 (0.079)	1.026 (0.034)	1.245 (0.097)	1.03 (0.042)

Note. For each social health measure, a separate zero-inflated negative binomial model is presented (Model 1 for social connectedness and Model 2 for perceived social support) predicting current suicidal ideation presence and severity at baseline. The threshold for significance is set to p <.01. Estimates are presented as odds ratios for ideation presence (zero inflation part) and rate ratios for ideation severity and are coded such that odds ratios and rate ratios smaller than 1 indicate protective effects. Legend: SE, Standard Error; [#], Social Health Measure corresponds to Social Connectedness in Model 1 and to Perceived Social Support in Model 2; [•], p<.05 (nominal effect); significant effects: *, p<.01; **, p<.001.

Table S4

Zero-inflated negative binomial models evaluating the effect of, respectively, social connectedness and perceived social support by sex on prospectively measured current suicidal ideation (sensitivity analysis of principal models presented in Table 4).

	**Social Health Measure = Social Connectedness**		**Social Health Measure = Perceived Social Support**	
Dependent Variable	Prospectively Measured Current Suicidal Ideation			
Model	Sensitivity Analysis Model 1-3		Sensitivity Analysis Model 2-3	
Model Component	Ideation Presence<br>OR (SE)	Ideation Severity<br>RR (SE)	Ideation Presence<br>OR (SE)	Ideation Severity<br>RR (SE)
Time in days (log-transformed)	0.455•<br>(0.876)	0.929<br>(0.067)	0.404•<br>(1.108)	0.94<br>(0.057)
#**Social Health Measure**	0.353<br>(1.938)	0.706**<br>(0.056)	0.134**<br>(4.315)	0.656**<br>(0.057)
Age	0.892•<br>(0.062)	0.964<br>(0.021)	0.925<br>(0.055)	0.987<br>(0.021)
Sex (Male vs Female)	0.267<br>(3.405)	0.765<br>(0.247)	0.102•<br>(8.795)	0.913<br>(0.3)
Depression Severity	0.808*<br>(0.093)	1.109*<br>(0.035)	0.775•<br>(0.162)	1.068•<br>(0.031)
Physical Illness	5.063*<br>(0.103)	0.95<br>(0.109)	2.654**<br>(0.037)	0.991<br>(0.037)
#**Social Health Measure***Physical Illness	0.862<br>(0.118)	0.977<br>(0.012)	0.68*<br>(0.181)	1.017<br>(0.016)
#**Social Health Measure***Sex (Male vs Female)	1.028<br>(0.244)	1.396*<br>(0.161)	0.669<br>(0.575)	1.345<br>(0.211)

Note. For each social health measure, a zero-inflated negative binomial model is presented predicting prospectively measured suicidal ideation presence and severity. The threshold for significance is set to $p < .01$. These models are fit to evaluate the robustness of the perceived social support by physical illness interaction effect found in the principal analysis (Table 4) to a perceived social support by sex interaction. Estimates are presented as odds ratios for ideation presence (zero inflation part) and rate ratios for ideation severity and are coded such that odds ratios and rate ratios smaller than 1 indicate protective effects. Legend: SE, Standard Error; #, Social Health Measure corresponds to Social Connectedness in Models 1-1 to 1-3 and to Perceived Social Support in Models 2-1 to 2-3; •, $p<.05$ (nominal effect); significant effects: *, $p<.01$; **, $p<.001$.
